# Colonization and diversification of the *Euphorbia* species (sect. *Aphyllis* subsect. *Macaronesicae*) on the Canary Islands

**DOI:** 10.1038/srep34454

**Published:** 2016-09-29

**Authors:** Ye Sun, Yanshu Li, Carlos Fabián Vargas-Mendoza, Faguo Wang, Fuwu Xing

**Affiliations:** 1 Guangdong Key Laboratory for Innovative Development and Utilization of Forest Plant Germplasm, College of Forestry and Landscape Architecture, South China Agricultural University, Guangzhou 510642, China; 2 Key Laboratory of Plant Resources Conservation and Sustainable Utilization, South China Botanical Garden, Chinese Academy of Sciences, Guangzhou 510650, China; 3 University of Chinese Academy of Sciences, Beijing 100049, China; 4 Escuela Nacional de Ciencias Biológicas-Instituto Politécnico Nacional, Prolongación de Carpio y Plan de Ayala S/N, 11340 México D. F, Mexico

## Abstract

Diversification between islands and ecological radiation within islands are postulated to have occurred in the *Euphorbia* species (sect. *Aphyllis* subsect. *Macaronesicae*) on the Canary Islands. In this study, the biogeographical pattern of 11 species of subsect. *Macaronesicae* and the genetic differentiation among five species were investigated to distinguish the potential mode and mechanism of diversification and speciation. The biogeographical patterns and genetic structure were examined using statistical dispersal-vicariance analysis, Bayesian phylogenetic analysis, reduced median-joining haplotype network analysis, and discriminant analysis of principal components. The gene flow between related species was evaluated with an isolation-with-migration model. The ancestral range of the species of subsect. *Macaronesicae* was inferred to be Tenerife and the Cape Verde Islands, and Tenerife-La Gomera acted as sources of diversity to other islands of the Canary Islands. Inter-island colonization of *E. lamarckii* among the western islands and a colonization of *E. regis-jubae* from Gran Canaria to northern Africa were revealed. Both diversification between islands and radiation within islands have been revealed in the *Euphorbia* species (sect. *Aphyllis* subsect. *Macaronesicae*) of the Canary Islands. It was clear that this group began the speciation process in Tenerife-La Gomera, and this process occurred with gene flow between some related species.

The flora of the Macaronesian biogeographical region, including five archipelagos in the Atlantic Ocean (Azores, Maderia, Cape Verde, Selvagens Islands, and Canary Islands), is exceptionally rich and diverse, with a high degree of endemism, especially in the Canary Islands^1^,^2^. The Canarian archipelago is composed of seven main islands located next to the north-western part of the African continent. All of the islands are volcanic and oceanic in origin, with the ages decreasing from east to west; Fuerteventura is nearest to the continent, and La Palma is farthest from the mainland^2^.

The biogeographical affinities of the Canarian flora are diverse. Regarding the current endemic plants, 35% have Mediterranean affinities, 25% have relationships with northwest Africa, 22% are related to more distant regions (especially eastern and southern Africa and the New World), and 18% are derived from Macaronesian ancestors^3^. Distinct episodes of colonization for the Canary Islands have been revealed from western Mediterranean and northern Africa, coinciding with the successive waves of major geological and climatic changes in these regions^4^,^5^,^6^. Stepping stone colonization with a direction from older to younger islands is the simplest model of colonization within archipelagos^7^. This dispersal model, with east-west migration, has been revealed for some organisms across the Canarian archipelago^2^; however, this is not the only colonization mode, and several authors have discussed a predominant model of colonization of plants with the central Canary Islands as centres of dispersal^8^,^9^,^10^,^11^.

The endemics of the Canarian archipelago include taxa that have undergone diversification between islands but remain in similar ecological zones^12^,^13^ and groups that have radiated rapidly into distinct ecological zones^14^. Diversification between islands and radiation within islands have been proposed as prominent modes for the evolution of endemic species in the Canarian archipelago^13^,^14^. Although radiation to distinct habitats within islands may give support for sympatric speciation, allopatric speciation is very recurrent within the same Canarian islands, for example, due to lava flows or giant landslides^2^,^10^.

Succulent and dendroid *Euphorbia* species are characteristic of thermophilous and xerophilous vegetation that constitute a typical feature of the arid and sub-arid Canary landscape^15^. The *Euphorbia* species endemic to the Macaronesian region had been treated as the group “*Canariensis et Mediterraneae*” under sect. *Tithymalus* Boiss.^16^, which was subsequently proved to be polyphyletic and belonged to several colonization groups^17^. In recent molecular phylogenetic studies, *Euphorbia* sect. *Aphyllis* subsect. *Macaronesicae* Molero & Barres is defined to accommodate the Macaronesian species, including *E. anachoreta* Svent., *E. aphylla Brouss.* ex Willd., *E. atropurpurea* Brouss. ex Willd., *E. berthelotii* Bolle ex Boiss., *E. bourgeana* J. Gay ex Boiss., *E. bravoana* Svent., *E. lamarckii* Sweet, *E. pedroi* Molero & Rovira, *E. piscatoria* Aiton, *E. regis-jubae* Webb & Berthel., and *E. tuckeyana* Steud. ex Webb.^18^,^19^. These species have a nearly endemic distribution in Macaronesia (except for the Azores), the southern coast of Portugal, and the Atlantic coast of Morocco and Western Sahara and are sister-related to African-Arabian species in *Euphorbia* sect. *Aphyllis* subsect. *Africanae* Molero & Barres^18^,^19^. For the 11 Macaronesian species, *E. anachoreta, E. pedroi, E. piscatoria* and *E. tuckeyana* live in Selvagens, the Iberian Peninsula, Madeira, and Cape Verde, respectively. The other Macaronesian species are in the Canary Islands, where *E. aphylla* occurs in the central islands (La Gomera, Tenerife, Gran Canaria), *E. atropurpurea* occurs in Tenerife, *E. bourgeana* occurs in Tenerife and La Gomera, *E. berthelotii* and *E. bravoana* occur in La Gomera, *E. lamarckii* occurs in the western islands (Tenerife, La Gomera, La Palma, and EI Hierro), and *E. regis-jubae* occurs in the eastern islands (Gran Canaria, Lanzarote, and Fuerteventura) and the Atlantic coast of Morocco^15^. These Canarian species have high morphological similarity and their taxonomic classification is easy to confuse, especially for *E. lamarckii* and *E. regis-jubae*^15^.

Diversification between islands and adaptive ecological radiation are postulated to occur in the *Euphorbia* sect. *Aphyllis* subsect. *Macaronesicae* on the Canary Islands^18^. However, the ancestral area of this group was unclear, and the genetic patterns associated with the diversification and speciation of this group within the Canarian archipelago have not been addressed. In this study, we intend to corroborate the possible location of origin of subsect. *Macaronesicae* and to identify the factors (dispersal, extinction, vicariance) that are responsible for the current distribution pattern. Once we establish the ancestral area and the species that occurred there, we expect to reveal the process of colonization and diversification of this group, the genetic differentiation of these closely related species, and the potential role of inter-island colonization and ecological shifts in the evolution of endemic species by investigating the genetic diversity of *E. atropurpurea, E. berthelotii, E. lamarckii*, and *E. regis-jubae* across the Canary Islands with chloroplast DNA sequences and nuclear SSR (simple sequence repeat) markers from population-level sampling.

## Results

### Phylogenetic analysis and divergence time estimation

We conducted our phylogenetic analysis based on concatenated sequences. A phylogenetic tree (Fig. S1, Supporting Information) was constructed based on the concatenated sequence of chloroplast tRNA-Leu, psbA-trnH, ndhF and nuclear ITS (Table S1, Supporting Information). The deeper node shows that the subfamily Euphorbioideae had separated from the other subfamilies of the Euphorbiaceae family 75.56 ± 6.97 (PP = 1.0) Ma ago in the Upper Cretaceous. The phylogenetic relationships of sect. *Aphyllis* (Fig. S2, Supporting Information) were partially resolved by including four more chloroplast genes (Table S1, Supporting Information). The 11 recognized species constituted subsect. *Macaronesicae* and had diverged from subsect. *Africanae* 9.81 ± 1.05 (PP = 1.0) Ma ago in the Miocene. The first divergence within the *Macaronesicae* group was 6.92 ± 1.55 (PP = 1.0) Ma ago, when *E. tuckeyana* separated from the rest of the group; after that, *E. lamarckii* diverged 1.93 ± 0.98 (PP = 1.0) Ma ago in the Pleistocene. The other species of the *Macaronesicae* group split into three subgroups, but they had very low support according to posterior probability.

### Biogeographical history

**Figure 1:**
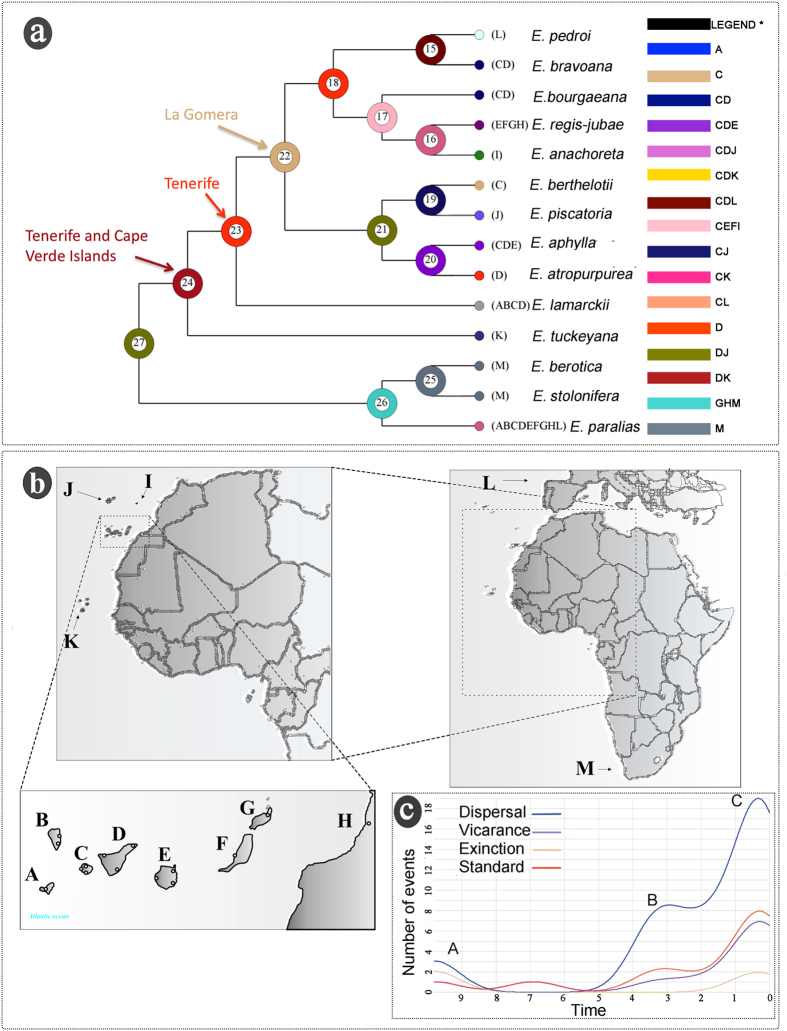
(**a**) Phylogenetic tree and results of statistical dispersal-vicariance analysis (S-DIVA) based on DNA sequences of eight chloroplast genes and nuclear ITS. The most likely areas in nodes 22, 23 and 24 are shown; the letters next to each taxon show its current distribution area as defined in the legend; the letters in the legend show the possible ancestral ranges at different nodes, which are represented by different colours; (**b**) The thirteen geographical areas defined in S-DIVA: A = El Hierro; B = La Palma; C = La Gomera; D = Tenerife; E = Gran Canaria; F = Fuerteventura; G = Lanzarote; H = Morocco and North of Africa; I = Selvagens Islands; J = Madeira; K = Cape Verde Islands; L = South of Iberian Peninsula; and M = South Africa; (**c**) The time-event curves with the most important biogeographical events in the group (A–C) showing the dispersal (blue line), vicariance (purple line), and extinction (light pink line) events; the orange line is the number of nodes on the time. All of the maps were drawn with the free software DIVA-GIS v7.5 (http://www.diva-gis.org/download).

The results of statistical dispersal-vicariance analysis (S-DIVA) suggested 31 dispersal, eight vicariance and two extinction events for all 14 species analysed, and 21 dispersal, six vicariance and two extinction events for subsect. *Macaronesicae*. The ancestral range of subsect. *Macaronesicae* at node 24 was Tenerife and Cape Verde Islands, where one vicariance event was inferred for the species *E. tuckeyana* (Fig. 1a,b). At node 23, Tenerife is the ancestral area for species inhabiting the Canary Islands (areas from A to G), Selvagens Islands (I), and Madeira (J). The other nodes showed very low support, which does not allow us to clearly define an ancestral area. Dispersal events (for all 14 species) mainly occurred at three points in time: 9.34 Ma ago (Point A in Fig. 1c), 3.2 Ma ago (Point B in Fig. 1c) and less than a million years ago (Point C in Fig. 1c). The global pattern was that four speciation events occurred in La Gomera and Tenerife (*E. atropurpurea, E. berthelotii, E. bravoana,* and *E. bourgaeana*), and one more speciation event occurred in South Africa (*E. berotica-E. stolonifera*).

### Genetic differentiation and population structure

**Figure 2:**
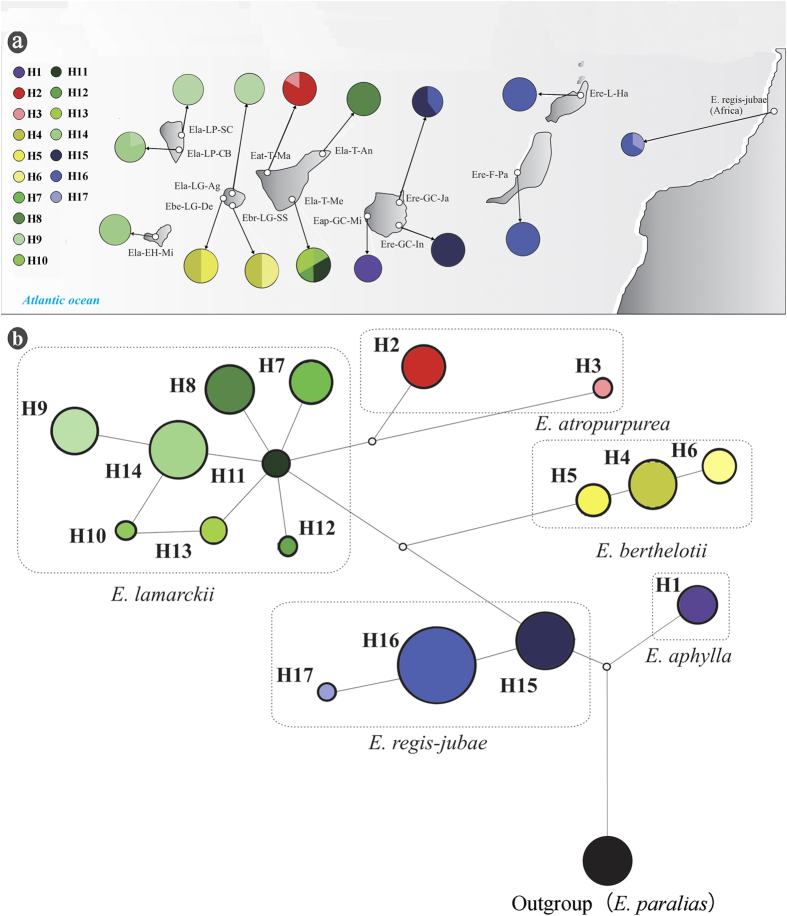
(**a**) Localities and chloroplast haplotypes of the sampled populations of *E. lamarckii, E. regis-jubae, E. berthelotii, E. atropurpurea* and *E. aphylla* on the Canary Islands. The populations correspond to the names in Table 1; (**b**) A reduced median-joining network of chloroplast haplotypes showed their relationships. All of the maps were drawn with the free software DIVA-GIS v7.5 (http://www.diva-gis.org/download).

**Figure 3:**
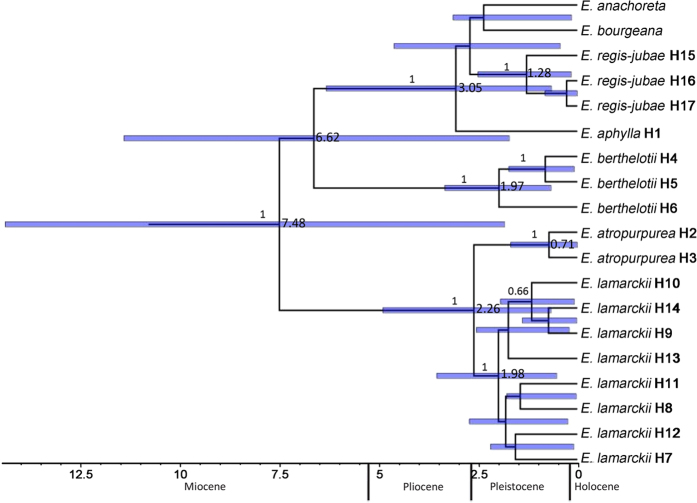
Bayesian phylogenetic tree constructed for the chloroplast haplotypes revealed in *E. lamarckii, E. regis-jubae, E. berthelotii, E. atropurpurea* and *E. aphylla*, together with *E. anachoreta* and *E. bourgeana*. The posterior probability is shown above the branches. Blue bars represent confidence intervals of divergence time, which are shown to the right of the internal nodes.

The chloroplast genetic diversity of *E. aphylla, E. atropurpurea, E. berthelotii, E. lamarckii*, and *E. regis-jubae* across the Canary Islands was investigated at the population-level, although the population sampling of *E. aphylla* and *E. atropurpurea* was limited. A total of 17 haplotypes were revealed in the five species (Fig. 2a). On the Canary Islands, eight populations were fixed by a single haplotype, and six populations were polymorphic; the Ela-T-Me population of *E. lamarckii* on Tenerife possessed the highest number of haplotypes. The relationships of these chloroplast haplotypes were revealed through reduced median-joining network analysis (Fig. 2b). Five haplogroups were detected and corresponded to five species very well. Haplotype H1 was found only in *E. aphylla*, H2 and H3 were found in *E. atropurpurea*, and H4, H5 and H6 were found in *E. berthelotii*. Haplotypes H7–H14 were found in *E. lamarckii*, and H15, H16 and H17 were found in *E. regis-jubae*. No common haplotypes were shared between any pairs of species. Clustering of haplotypes based on phylogeny (Fig. 3) showed a clade containing *E. atropurpurea* and *E. lamarckii*, with a posterior probability of 1.0, that separated 7.48 ± 2.55 Ma ago. By contrast, a clade composed of haplotypes of *E. berthelotii* (PP = 1.0) diverged 6.62 ± 1.99 Ma ago, and a sister group including *E. anachoreta, E. bourgeana, E. regis-jubae* and *E. aphylla* haplotypes formed (PP = 1.0) 3.05 ± 1.71 Ma ago. More recently, a node grouping *E. regis-jubae* haplotypes emerged at 1.28 ± 0.53 Ma ago (PP = 1.0).

When using chloroplast data from *E. lamarckii, E. regis-jubae, E. berthelotii* and *E. atropurpurea*, the values of Tajima’s D and Fu’s F_S_ were positive but did not differ significantly from zero, and the test of goodness-of-fit rejected the demographic expansion model (P_SSD_ = 0.011; P_Rag_ = 0.002) but not the spatial expansion model (P_SSD_ = 0.283; P_Rag_ = 0.210).

The nuclear genetic diversity and allelic richness in each population were 0.284–0.757 and 2.516–6.985 (Table 1), respectively. The Ela-T-Me population of *E. lamarckii* on Tenerife possessed the highest genetic diversity and allelic richness, whereas the Eat-T-Ma population of *E. atropurpurea* on Tenerife had the lowest genetic diversity and allelic richness. The number of alleles revealed per locus was 11–23, and the observed heterogeneities were 0.407–0.596 (Table 2). Except for locus E42, the inbreeding coefficient (*F*_*IS*_) was significantly different from zero (P < 0.05). The genetic differentiation (*F*_*ST*_) estimated after excluding null alleles was 0.160–0.314. There was no evidence that microsatellites were affected by selection (Fig. S3 and Supplementary Information).

**Figure 4:**
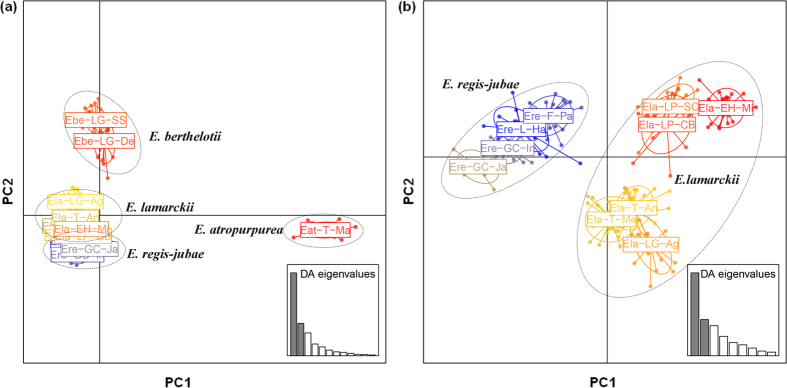
Population structure described by discriminant analysis of principal components (DAPC) based on nuclear microsatellites. Each colour corresponds to a single population, and ellipses with dashed lines represent species. (**a**) 13 populations of *E. lamarckii, E. regis-jubae, E. berthelotii* and *E. atropurpurea*; (**b**) 10 populations of *E. lamarckii* and *E. regis-jubae*.

The population structure was described using discriminant analysis of principal components (DAPC); the first axis distinguished *E. atropurpurea* from the other three species, and the second axis separated *E. berthelotii, E. lamarckii* and *E. regis-jubae* (Fig. 4a). In a further analysis including only *E. lamarckii* and *E. regis-jubae*, a clearer differentiation between *E. lamarckii* and *E. regis-jubae* was shown along the PC1 axis (Fig. 4b), and the PC2 axis further revealed a strong structure in *E. lamarckii* that split its eastern populations on La Palma and EI Hierro from the rest of the populations on La Gomera and Tenerife.

**Figure 5:**
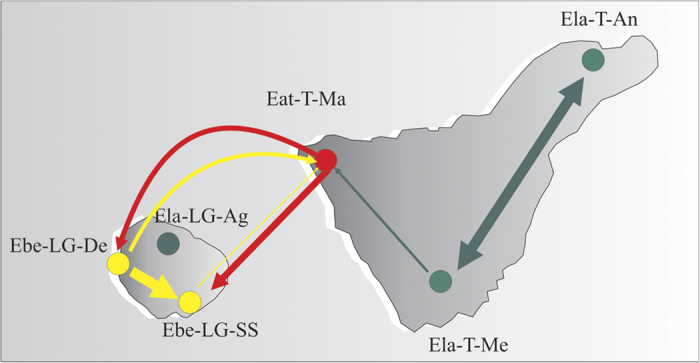
Gene flow estimation between populations on Tenerife and La Gomera based on nuclear microsatellites and chloroplast DNA sequences. Arrow widths indicate the magnitude of gene flow; arrow colours represent species from which the gene flow originates (red, *E. atropurpurea*; green, *E. lamarckii*; and yellow, *E. berthelotii*). All of the maps were drawn with the free software DIVA-GIS v7.5 (http://www.diva-gis.org/download).

No evidence of gene flow was observed between the *E. lamarckii* and *E. berthelotii* species; however, possible evidence of significant gene flow was detected between the species pairs of *E. atropurpurea* and *E. lamarckii*, and *E. atropurpurea* and *E. berthelotii* (Figs 5 and S4). The maximum-likelihood estimates (MLE) and 95% highest probability density (HPD) intervals of gene flow rate are shown in Supplementary Table S2. Only a unidirectional migration rate from *E. lamarckii* to *E. atropurpurea* was detectable; the migration rate per generation from the Ela-T-Me population to the Eat-T-Ma population was 0.0312 (HPD: 0.0000–0.9351). Bidirectional gene flow was revealed between *E. atropurpurea* and *E. berthelotii*; the migration rates from the Eat-T-Ma population to the Ebe-LG-SS population and the Ebe-LG-De population were 0.1836 (HPD: 0.0000–0.8390) and 0.1262 (HPD: 0.0000–0.8955), respectively, whereas the migration rate from the Ebe-LG-SS population to the Eat-T-Ma population was 0.0045 (HPD: 0.0000–0.6707), and the migration rate from the Ebe-LG-De population to the Eat-T-Ma population was 0.1025 (HPD: 0.0000–0.8677).

## Discussion

In the present study, we were able to determine the area of origin for one of the most characteristic groups in the region of Macaronesia, namely the genus *Euphorbia* sect. *Aphyllis* subsect. *Macaronesicae*. Our results showed that Tenerife and the Cape Verde Islands played the most important role in the origin and speciation of subsect. *Macaronesicae*; in particular, Tenerife - La Gomera was the area of origin of these species in the archipelago of the Canary Islands. Tenerife initially existed as three separate islands (Roque del Conde, Teno, and Anaga)^20^. Palaeo-island Roque del Conde in the southwest of Tenerife is very likely to have received migrants from Cape Verde due to the very close distance. La Gomera and the three palaeo-islands of Tenerife have been geologically stable since the Pliocene^9^ and thus could act as repositories and stepping stones for the Macaronesian plant group^21^. We further discovered that dispersal events (Fig. 1c) mainly occurred at three timepoints, at 9.34 Ma, 3.2 Ma and 1 Ma, in which the first and third periods coincided with the emergence of La Gomera and EI Hierro. The present study revealed that populations on Tenerife, Gran Canaria, and La Gomera generally harboured the highest level of nuclear variation and possessed diverse chloroplast haplotypes (Table 1 and Fig. 2a), highlighting the important role of the central Canary Islands as cradles of biodiversity and as sources of diversity for other islands and for the mainland^9^,^10^,^22^,^23^. The estimation of age for the disjunction between subsect. *Macaronesicae* and subsect. *Africanae* predated the formation of the Sahara (approximately 6 Ma) and thus agrees well with a climate-driven vicariance explanation, as has been reported in other groups such as the genera *Canarina, Plocama, Campylanthus, Camptoloma*, and *Hypericum*^4^,^22^.

Our results revealed a relatively rapid evolution of subsect. *Macaronesicae* on the Canary Islands (Figs 3 and S2), which was consistent with a previous study that showed that *Euphorbia* sect. *Aphyllis* is a group that has diverged much faster than expected^4^. The Bayesian tree of subsect. *Macaronesicae* produced in the present study was partially consistent with the results of previous studies^4^,^18^,^19^; one of the similarities was the formation of a clade that consisted of *E. lamarckii* and another composed of *E. regis-jubae*. However, the species that were grouped with these two species were not entirely similar to the species groupings that were reported in previous studies^4^,^18^. What has happened in some species and the patterns between related species are discussed later because inter-island colonization seems to have played a major role in the diversification of subsect. *Macaronesicae*.

The genetic divergence between the “eastern” islands and the “western” islands has been revealed in numerous groups such as *Canarina canariensis*^9^ and *Phoenix canariensis*^24^. In the present study, the west-east split corresponded to species differentiation between *E. regis-jubae* and *E. lamarckii*, which could be interpreted as an inter-island colonization and diversification in the archipelago^10^,^25^. *E. regis-jubae* and *E. lamarckii* are xerophilous and thermophilous species with a marked invasive capacity and appear to live in similar ecological conditions because both are very common constituents of the coastal belt vegetation at low elevations in the arid and semi-arid infracanarian bioclimatic belt^15^. The two species possessed roughly equal genetic diversity within populations in the present study. The two species have subtle morphological differences such as the sizes of bracts, and the subordination of *E. regis-jubae* to *E. lamarckii* as a subspecies was once the accepted view^15^. Their morphological variation might simply be a consequence of divergence through gradual transformation after inter-island colonization and finally the accumulation and harbouring of considerable genetic diversity through mutation and recombination^26^.

The inter-island colonization routes of the two widespread species could be inferred based on the chloroplast haplotype relationships. For *E. lamarckii*, the ancient haplotype (H11) was revealed in the Ela-T-Me population on Tenerife, and the haplotypes from La Gomera, La Palma and EI Hierro were paraphlyletic to the haplotypes from Tenerife, indicating that this species dispersed from Tenerife to La Gomera, La Palma and EI Hierro, which was consistent with an earlier study^9^ and was further supported by S-DIVA analysis that suggested that Tenerife was the ancestral area for species that inhabit the Canary Islands. Similar to the genetic diversity pattern reported in *Phoenix canariensis*^24^, a split between the populations of *E. lamarckii* from La Palma and EI Hierro and from La Gomera and Tenerife was revealed by nuclear microsatellites; this isolation was reasonable when taking into account the fact that La Gomera and Tenerife are much older than La Palma and EI Hierro. For *E. regis-jubae*, the ancient haplotype (H15) was found on Gran Canaria. Three individuals of this species in Morocco possessed the derived haplotypes H16 and H17, indicating a colonization of this species from Gran Canaria to northern Africa via Fuerteventura and Lanzarote. The colonization of the African continent suggested that the Canary Islands may play a role as refugia and a supply source for continental biodiversity^5^,^23^. The fact that the common chloroplast haplotype H16 was shared across the central and eastern islands suggested seed dispersal among islands because the chloroplast genome is transmitted maternally in most angiosperms. Considering other evidence for inter-island gene flow between *E. atropurpurea* on Tenerife and *E. berthelotii* on La Gomera, inter-island colonization was proposed to play a prominent role in the diversification of the *Euphorbia* sect. *Aphyllis* subsect. *Macaronesicae* on the Canary Islands.

The present study revealed that no haplotypes were shared between any pairs of species and that the haplotype phylogenetic tree (Fig. 3) could mirror the species’ history. However, possible evidence of significant gene flow was detected between species pairs of *E. atropurpurea* and *E. lamarckii*, and *E. atropurpurea* and *E. berthelotii*. Gene flow between species may be a cause for the incongruence between phylogeny trees constructed based on the species and haplotype levels and for the low supportiveness of some nodes in the former (Fig. 1a) because it combined chloroplast and nuclear sequences. When natural selection drives splitting divergence in the presence of migration^27^, the species genome may remain selectively porous to gene flow even long after speciation^28^. Although difficult to detect, hybridization and introgression are more prone to happen in island-related taxa and are proposed to play important roles in the evolution of Macaronesian plants^29^. Speciation via adaptive radiation that is driven by selection within markedly different ecological zones is common within islands^14^. High volcanoes with sharp elevation gradients in climate and the complex topographies and secondary eruptions in the central and western islands of Canarian archipelago may facilitate within-island diversification^2^,^25^.

A clear link between climatic conditions and adaptation in succulent *Euphorbia* was revealed in Madagascar^30^. The divergence between *E. atropurpurea* and *E. lamarckii* on Tenerife and *E. berthelotii* and *E. lamarckii* on La Gomera might be cases of within-island radiation because they occurred at different elevation zones of climate. Unlike *E. lamarckii* occupying xerophytic habitats in the basal vegetation belts of the Canary Islands, *E. atropurpurea* and *E. berthelotii* preferred the mesophitic and sub-hygrophytic areas at moderate elevations on Tenerife and La Gomera^17^,^31^. The level of genetic variation within populations of *E. atropurpurea* was obviously lower than within populations of *E. lamarckii* (Table 1), which provided further evidence of adaptive divergence^26^; however, caution must be exercised in interpreting this result because we sampled only one population of *E. atropurpurea*, and several important populations in the palaeo-island of Teno and Roque del Conde (Adeje) were not included in this study; these ancient areas could have acted as refugia in the central Canary islands and could be considered as separate units in the phylogeographical history of Tenerife^9^. Although no evidence of gene flow was revealed between *E. berthelotii* and *E. lamarckii* on La Gomera, gene flow was detected between species pairs of *E. atropurpurea* and *E. lamarckii* on Tenerife; thus, the present study may provide an example of adaptive divergence in the face of gene flow.

## Material and Methods

### Data collection

A total of 14 natural populations, including six populations of *Euphorbia lamarckii*, four populations of *E. regis-jubae*, two populations of *E. berthelotii*, one population of *E. atropurpurea*, and one population of *E. aphylla* (Table 1 and Fig. 2a), as well as two individuals of *E. anachoreta* and *E. bourgeana* were collected on the Canary Islands during 2011 and 2012. Vouchers were deposited at the herbarium of the South China Botanical Garden (formerly the South China Institute of Botany), Chinese Academy of Sciences (IBSC). One population of *E. paralias* L. was sampled as an outgroup. Three individuals of *E. regis-jubae* in Morocco were collected and provided by Professor Paul E. Berry (University of Michigan).

Thirteen populations were genotyped with 10 polymorphic nuclear microsatellites developed in *E. lamarckii*^32^. *E. aphylla* was not genotyped with SSR due to the very small sample size. Forward primers were labelled at the 5′-end with the fluorochromes TAMRA, HEX, 6-FAM and ROX, and polymerase chain reaction (PCR) products were separated and visualized on an ABI-377 fluorescence sequencer (Applied Biosystems, Carlsbad, CA, USA).

Four non-coding chloroplast regions including the trnL intron, trnL-trnF, trnS-trnG, and psbM-trnD were sequenced. The TrnL intron and trnL-trnF were amplified and sequenced with universal primer pairs of c and d, e and f as described by Taberlet *et al*.^33^. TrnS-trnG and psbM-trnD were amplified and sequenced by primer pairs of trnS^GCU^ and 5′trnG2S, psbMF and trnD^GUC^R^34^.

### Statistical analyses

#### Phylogenetic analysis and divergence time estimation

We carried out the phylogenetic reconstruction and calibrated the divergence time step by step. First, the node time of the subfamilies of Euphorbiaceae was estimated through phylogenetic analysis of a set of DNA sequences of 16 species within three subfamilies (Acalyphoideae, Crotonoideae, and Euphorbioideae). DNA sequences of three chloroplast genes (tRNA-Leu, psbA-trnH and ndhF) and nuclear ITS were retrieved from GenBank (see Table S1, Supporting Information for accessions). The incongruence length difference (ILD) test^35^ was performed with PAUP^36^ to evaluate the congruence of the partitioned datasets, and a P value of the ILD test was not lesser than 0.01, which suggested that combining the data improved or did not reduce the phylogenetic accuracy^37^. Homoplasy and substitution saturation were assessed with PAUP and DAMBE5^38^. Low homoplasy and little saturation were revealed for the concatenated sequences. Phylogenetic analyses were conducted with the multi-species coalescent approach implemented in BEAST v2.4.0^39^ using a calibrated Yule speciation process with the unlink option for tree, site and clock models in the partition panel. The substitution model of TN93 for chloroplast concatenated genes and HKY for ITS were selected based on the Akaike Information Criterion (AIC) by using the program MODELTEST v3.7^40^,^41^. The uncorrelated lognormal relaxed clock was used for both partitions. The multi-species coalescent approach was run using a constant population function with an autosomal nuclear ploidy for ITS and Y or mitochondrial for chloroplast genes. We used a run of 2 × 10^7^ generations for the MCMC search and sampled every 2000^th^ generation. It was considered to be a stationary state of the Markov chain when all of the parameters obtained an ESS (Effective Sampling Sizes) value of more than 200 as measured in the TRACER v1.6 program^42^. FIGTREE v.1.4.2^43^ was used to generate and visualize the resulting maximum clade credibility (MCC) chronograms. The nodes were calibrated according to the divergence time of the subfamily Euphorbiaceae (74.25 ± 7.35 Ma) provided in Magallón *et al*.^44^.

Subsequently, four more chloroplast genes were included, and a total of eight chloroplast genes and nuclear ITS (see Table S1, Supporting Information for accession) of 14 species were used in phylogenetic reconstruction with the multi-species coalescent approach as in the previous step. The 14 species were from two sections of *Euphorbia* (sect. *Aphyllis* and sect. *Paralias*), including all 11 species of sect. *Aphyllis* subsect. *Macaronesicae*, two species of sect. *Aphyllis* subsect. *Africanae (E. berotica* and *E. stolonifera*), and *E. paralias* of sect. *Paralias*. A calibrated Yule prior was used with GTR substitution models for both partitions. Strict and uncorrelated lognormal clocks were used for the chloroplast and nuclear genes, respectively. This combination gave the highest value of ESS. The divergence time obtained in the former step (node for *Africanae* 3.06 ± 0.97 Ma; node for *Macaronesicae* 7.98 ± 1.22 Ma; and node for *Aphylli*s 9.05 ± 1.28 Ma) was used as prior to calibrate this group.

Finally, we made a phylogenetic analysis of the haplotypes revealed in this study using a multi-species coalescent approach with the same parameters defined in the previous two datasets. The haplotypes were determined with DNASP version 5.0^45^ from DNA sequences (NCBI accession No. KR188519–KR188875) obtained in this study. The calibrated Yule prior, GTR substitution model, and uncorrelated lognormal relaxed clock were chosen. In the dating of haplotypes, a nested-dating partitioned method^9^,^23^ was used to apply different tree priors for inter- and intra-specific relationships, and the date estimation (6.92 ± 1.55 Ma for subsect. *Macaronesicae*) obtained in the second step (Fig. S2, Supporting Information) was taken to calibrate the haplotype divergence within populations.

#### Biogeographical and phylogeographical analyses

The biogeographical history of subsect. *Macaronesicae* was investigated based on eight chloroplast genes and nuclear ITS using the S-DIVA approach implemented in RASP v.1.2^46^. Thirteen geographical areas were defined: A = El Hierro; B = La Palma; C = La Gomera; D = Tenerife; E = Gran Canaria; F = Fuerteventura; G = Lanzarote; H = Morocco and North of Africa; I = Selvagens Islands; J = Madeira; K = Cape Verde Islands; L = South of Iberian Peninsula; and M = South Africa (Fig. 1b). We used the following settings: maximum area at each node = 2; allow extinction (slow); allow reconstruction (slow); use ancestral ranges of condensed trees^46^. Additionally, we calculated time-event curves using four functions for the dispersion, extinction, and vicariance events and the total number of biogeographical events over time^46^.

A reduced median-joining network was constructed with NETWORK v4.5.1.6^47^ to resolve the haplotype relationships. Neutrality tests were performed by calculating the significance of Tajima’s D^48^ and Fu’s F_S_^49^. The distribution of the observed number of differences between pairs of haplotypes was examined to assessed demographic expansion with ARLEQUIN version 3.0^50^. Harpending’s Raggedness index^51^ and the sum of square deviations (SSD) between the observed and expected mismatch were used as test statistics for goodness of fit to the expansion model; a significant P value from 10,000 bootstrap replicates rejected the fit of the data to the expansion model.

#### Population structure and gene flow

The software MICRO-CHECKER^52^ was used to identify and correct genotyping errors in the microsatellite data. No evidence of scoring error due to stuttering and large allele dropout was found. Population genetic parameters including the number of alleles detected (*A*), allelic richness (*A*_*R*_) rarefied to the smallest sample size of 11 diploid individuals per population, observed heterozygosity (*H*_*O*_), gene diversity within populations (*H*_*S*_), gene diversity in the total population (*H*_*T*_), inbreeding coefficient (*F*_*IS*_) and genetic differentiation among populations (*F*_*ST*_) were calculated at each locus using FSTAT version 2.9.3^53^. The frequencies of null alleles were evaluated with the program FREENA^54^ using 10,000 replicate simulations, and a refined estimation of population differentiation (*F*_*ST*_) was obtained after excluding null alleles. The Hardy-Weinberg equilibrium and genotypic disequilibrium were tested with Bonferroni corrections^55^. An *F*_*ST*_-outlier approach implemented in ARLEQUIN 3.5^50^ was used to test whether microsatellites were affected by selection. Population structure was described by discriminant analysis of principal components (DAPC) with the ADEGENET package in R^56^. DAPC does not rely on a particular population genetics model and is thus free of assumptions about the Hardy-Weinberg equilibrium or linkage disequilibrium^56^. The genetic variation was partitioned by analysis of molecular variance (AMOVA) using the program ARLEQUIN 3.5.

For *E. berthelotii, E. atropupurea*, and *E. lamarckii*, gene flow between their populations on La Gomera and Tenerife was inferred using the program IMA2^27^. Nine microsatellites with perfect dinucleotide repeats and chloroplast sequences were used in this analysis. The mutation rates for these microsatellites and chloroplast sequences were obtained from the earlier estimates^57^,^58^. A geometric heating model was used with a first heating parameter of 0.95 and a second heating parameter of 0.91. The maximum population size (4 Nu) was set to 100, and the upper bound of the priors for migration and divergence time (maximum time of population splitting) were set to m = 0.99 and t = 10.0, respectively. A total of 100 independent chains with burn-in durations of 1 million steps were run for the MCMC procedure^27^.

## Additional Information

**How to cite this article**: Sun, Y. *et al*. Colonization and diversification of the *Euphorbia* species (sect. *Aphyllis* subsect. *Macaronesicae*) on the Canary Islands. *Sci. Rep.* **6**, 34454; doi: 10.1038/srep34454 (2016).

## Supplementary Material

Supplementary Information

## Figures and Tables

**Table 1:** Location, sample size and gene diversity of the sampled populations of *Euphorbia* species on the Canary Islands.

Species	Island	Location/code	Voucher	Latitude	Longitude	Individuals (sequenced)	Haplotype	*A*_*R*_	*H*	*F*_*IS*_
*E. regis-jubae*	Lanzarote	Haria/Ere-L-Ha	Canary-Sun-95	29°07′	013°30′	24(6)	H16(6)	4.430	0.573	0.113**
	Fuerteventura	Pajara/Ere-F-Pa	Canary-Sun-92	28°19′	014°06′	24(6)	H16(6)	5.185	0.692	0.254**
	Gran Canaria	Ingenio/Ere-GC-In	Canary-Sun-113	27°56′	015°25′	24(6)	H15(6)	6.444	0.750	0.239**
	Gran Canaria	Jadin/Ere-GC-Ja	Canary-Sun-69	28°03′	015°27′	11(5)	H16(2),H15(3)	5.100	0.652	0.275**
*E. lamarckii*	Tenerife	Medano/Ela-T-Me	Canary-Sun-107	28°05′	016°33′	24(6)	H10(1),H11(2),H12(1),H13(2)	6.985	0.757	0.180**
	Tenerife	Anaga/Ela-T-An	Canary-Sun-86	28°33′	016°18′	24(6)	H8(6)	5.534	0.696	0.246**
	La Gomera	Agula/Ela-LG-Ag	Canary-Sun-104	28°11′	017°11′	24(5)	H7(5)	4.015	0.473	0.268**
	La Palma	Santa Cruz/Ela-LP-SC	Canary-Sun-100	28°42′	017°45′	26(5)	H9(1),H14(4)	5.501	0.646	0.113**
	La Palma	Cueva de Belmaco/Ela-LP-CB	Canary-Sun-75	28°43′	017°46′	19(5)	H9(5)	5.037	0.666	0.099*
	EI Hierro	Mirador de la Pena/Ela-EH-Mi	Canary-Sun-80	27°45′	018°00′	23(5)	H14(5)	5.415	0.635	0.130**
*E. berthelotii*	La Gomera	San Sebastian/Ebe-LG-SS	Canary-Sun-101	28°05′	017°07′	28(6)	H4(3),H6(3)	4.726	0.602	0.229**
	La Gomera	Degollada/Ebe-LG-De	Canary-Sun-76	28°05′	017°10′	20(6)	H4(3),H5(3)	5.861	0.708	0.251**
*E. atropurpurea*	Tenerife	Masca/Eat-T-Ma	Canary-Sun-88	28°18′	016°50′	22(6)	H2(5),H3(1)	2.516	0.284	0.147*
*E. aphylla*	Gran Canaria	Mirador de Balcon/Eap-GC-Mi	Canary-Sun-111	28°01′	015°47′	4(4)	H1(4)			

*Departure from Hardy-Weinberg (P < 0.05); **Departure from Hardy-Weinberg (P < 0.01).

**Table 2:** Characteristics of the 11 nuclear microsatellites used in this study.

Locus	*A*	*A*_*R*_	*H*_*O*_	*H*_*S*_	*H*_*T*_	*F*_*IS*_	Null allele frequency	*F*_*ST*_	*F*_*ST*_ after excluding null alleles
E86	19	10.843	0.500	0.672	0.906	0.248**	0.102	0.273	0.252
E90	14	7.539	0.481	0.570	0.806	0.159**	0.066	0.318	0.305
E89	22	9.881	0.596	0.718	0.882	0.170**	0.074	0.204	0.195
E95	19	9.483	0.591	0.639	0.890	0.070*	0.036	0.291	0.281
E92	14	8.809	0.563	0.625	0.889	0.091**	0.036	0.322	0.314
E42	11	5.681	0.490	0.470	0.679	−0.035	0.029	0.313	0.303
E59	12	5.925	0.407	0.513	0.673	0.173**	0.083	0.262	0.252
E65	16	8.642	0.463	0.647	0.883	0.295**	0.107	0.276	0.262
E97	23	10.770	0.430	0.760	0.907	0.428**	0.172	0.175	0.160
E78	15	8.926	0.496	0.640	0.867	0.233**	0.087	0.282	0.275
Overall		8.650	0.502	0.626	0.838	0.195**	0.079	0.271	0.260

*Departure from Hardy-Weinberg (P < 0.05); **Departure from Hardy-Weinberg (P < 0.01).

## References

[b1] Santos-Guerra A. Flora vascular nativa. In Naturaleza de las Islas Canarias. Ecología y conservación (eds Fernández-Palacios J. M. & Martin Esquivel J. L. ) 185–192 (Turquesa, Santa Cruz de Tenerife, 2001).

[b2] Juan C., Emerson B. C., Oromí P. & Hewitt G. M. Colonization and diversification: towards a phylogeographic synthesis for the Canary Islands. Tree 15, 104–109 (2000). 10675925 10.1016/s0169-5347(99)01776-0

[b3] Caujapé-Castells J. . The status of plant conservation on the Macaronesian archipelagos. In Proceedings of the 4th Global Botanic Gardens Congress (eds IUCN) 1–15 (Glasnevin, Dublin, Ireland, 2010).

[b4] Pokorny L. . Living on the edge: timing of Rand Flora disjunctions congruent with ongoing aridification in Africa. Front. Genet. 6, 154 (2015). 25983742 10.3389/fgene.2015.00154 PMC4416453

[b5] Carine M. A., Russell S. J., Santos-Guerra A. & Francisco-Ortega J. Relationships of the Macaronesian and Mediterranean floras: molecular evidence for multiple colonizations into Macaronesia and back-colonization of the continent in *Convolvulus* (Convolvulaceae). Am. J. Bot. 91, 1070–1085 (2004). 21653463 10.3732/ajb.91.7.1070

[b6] Kim S. C. . Timing and tempo of early and successive adaptive radiations in Macaronesia. PLoS ONE 3, e2139 (2008). 18478126 10.1371/journal.pone.0002139 PMC2367450

[b7] Emerson B. C. Evolution on oceanic island: molecular phylogenetic approaches to understanding pattern and process. Mol. Ecol. 11, 951–966 (2002). 12030975 10.1046/j.1365-294x.2002.01507.x

[b8] Goodson B. E., Santos-Guerra A. & Jansen R. K. Molecular systematic of *Descurainia* (Brassicaceae) in the Canary Islands: biogeographic and taxonomic implications. Taxon 55, 671–682 (2006).

[b9] Mairal M. . Palaeo-islands as refugia and sources of genetic diversity within volcanic archipelagos: the case of the widespread endemic *Canarina canariensis* (Campanulaceae). Mol. Ecol. 24, 3944–3963 (2015). 26096229 10.1111/mec.13282

[b10] Puppo P. . Molecular phylogenetics of *Micromeria* (Lamiaceae) in the Canary Islands, diversification and inter-island colonization patterns inferred from nuclear genes. Mol. Phylogenet. Evol. 89, 160–170 (2015). 25937559 10.1016/j.ympev.2015.04.017

[b11] Sanmartín I., van der Mark P. & Ronquist F. Inferring dispersal: a Bayesian approach to phylogeny-based island biogeography, with special reference to the Canary Islands. J. Biogeogr. 35, 428–449 (2008).

[b12] Böhle U. R., Hilger H. H. & Martin W. F. Island colonization and evolution of the insular woody habit in *Echium* L. (Boraginaceae).Proc. Natl. Acad. Sci. USA 93, 11740–11745 (1996). 8876207 10.1073/pnas.93.21.11740 PMC38128

[b13] Francisco-Ortega J., Jansen R. K. & Santos-Guerra A. Chloroplast DNA evidence of colonization, adaptive radiation, and hybridization in the evolution of the Macaronesian flora. Proc. Natl. Acad. Sci. USA 93, 4085–4090 (1996). 11607675 10.1073/pnas.93.9.4085 PMC39491

[b14] Kim S. C., Crawford D. J., Francisco-Ortega J. & Santos-Guerra A. A common origin for woody *Sonchus* and five related genera in the Macaronesian islands: molecular evidence for extensive radiation. Proc. Natl. Acad. Sci. USA 93, 7743–7748 (1996). 8755546 10.1073/pnas.93.15.7743 PMC38818

[b15] Molero J. & Rovira A. M. A note on the taxonomy of the Macaronesian *Euphorbia obtusifolia* complex (Euphorbiaceae). Taxon 47, 321–332 (1998).

[b16] Boissier E. *Euphorbia* L. sect. *Anisophyllum*. In Prodromus Systematis Naturalis Regni Vegetabilis, vol. 15(2), (ed. De Candolle A. P. ) 11–52 (Victor Masson & Fils, Paris, 1862).

[b17] Molero J., Garnatje T., Rovira A., Garcia-Jacas N. & Susanna A. Karyological evolution and molecular phylogeny in Macaronesian dendroid spurges (*Euphorbia* subsect. *Pachycladae*). Plant Syst. Evol. 231, 109–132 (2002).

[b18] Barres L., Vilatersana R., Molero J., Susanna A. & Galbany-Casals M. Molecular phylogeny of *Euphorbia* subg. *Esula* sect. *Aphyllis* (Euphorbiaceae) inferred from nrDNA and cpDNA markers with biogeographic insights. Taxon 60, 705–720 (2011).

[b19] Riina R. . A worldwide molecular phylogeny and classification of the leaf spurges, *Euphorbia* subgenus *Esula* (Euphorbiaceae). Taxon 62, 316–342 (2013).

[b20] Ancochea E., Hernán F., Huertas M. J., Brändle J. L. & Herrera R. A new chronostratigraphical and evolutionary model for La Gomera: Implications for the overall evolution of the Canarian Archipelago. J. Volcanol. Geoth. Res. 157, 271–293 (2006).

[b21] Fernández-Palacios J. M. . A reconstruction of Palaeo-Macaronesia, with particular reference to the long-term biogeography of the Atlantic island laurel forests. J. Biogeogr. 38, 226–246 (2011).

[b22] Mairal M., Pokorny L., Aldasoro J. J., Alarcon M. & Sanmartín I. Ancient vicariance and climate-driven extinction explain continental-wide disjunction in Africa: the case of the Rand Flora genus *Canarina* (Campanulaceae). Mol. Ecol. 24, 1334–1354 (2015). 10.1111/mec.13114 25688489

[b23] Patiño J. . Approximate Bayesian computation reveals the crucial role of oceanic islands for the assembly of continental biodiversity. Syst. Biol. 64, 579–589 (2015). 25713307 10.1093/sysbio/syv013

[b24] Saro I., Gonzalez-Perez M. A., Garcia-Verdugo C. & Sosa P. A. Patterns of genetic diversity in *Phoenix canariensis*, a widespread oceanic palm (species) endemic from the Canarian archipelago. Tree Genet. Genom. 11, 815 (2015).

[b25] Meimberg H. . Molecular evidence for adaptive radiation of *Micromeria* Benth. (Lamiaceae) on the Canary Islands as inferred from chloroplast and nuclear DNA sequences and ISSR fingerprint data. Mol. Phylogenet. Evol. 41, 566–578 (2006). 16839782 10.1016/j.ympev.2006.05.037

[b26] Stuessy T. F. . Anagenetic evolution in island plants. J. Biogeogr. 33, 1259–1265 (2006).

[b27] Hey J. Isolation with migration models for more than two populations. Mol. Biol. Evol. 27, 905–920 (2010). 19955477 10.1093/molbev/msp296 PMC2877539

[b28] Bull V. . Polyphyly and gene flow between non-sibling *Heliconius* species. BMC Biol. 4, 11 (2006). 16630334 10.1186/1741-7007-4-11 PMC1481601

[b29] Herben T., Suda J. & Muncliger P. The ghost of hybridization past: niche pre-emption is not the only explanation of apparent monophyly in island endemics. J. Ecol. 93, 572–575 (2005).

[b30] Evans M. . Insights on the evolution of plant succulence from a remarkable radiation in Madagascar (*Euphorbia*). Syst. Biol. 63, 698–711 (2014). 10.1093/sysbio/syu035 24852061

[b31] Del-Arco M. J. . Bioclimatology and climatophilous vegetation of Gomera (Canary Islands). Ann. Bot. Fenn. 46, 161–191 (2009).

[b32] Li Y. S., Sun Y., Wang F. G. & Xing F. W. Isolation and characterization of microsatellite loci in *Euphorbia lamarckii* Sweet (Euphorbiaceae) from the Canary Islands. Conserv. Genet. Resour. 6, 313–314 (2014).

[b33] Taberlet P., Gielly L., Pautou G. & Bouver J. Universal primers for amplification of three non-coding regions of chloroplast DNA. Plant Mol. Biol. 17, 1105–1109 (1991). 1932684 10.1007/BF00037152

[b34] Shaw J. . The tortoise and the hare II: relative utility of 21 noncoding chloroplast DNA sequences for phylogenetic analysis. Am. J. Bot. 92, 142–166 (2005). 21652394 10.3732/ajb.92.1.142

[b35] Farris J. S., Kallersjo M., Kluge A. G. & Bult C. Constructing a significance test for incongruence. Syst. Biol. 44, 570–572 (1995).

[b36] Swofford D. L. PAUP* (Phylogenetic Analysis Using Parsimony* and other methods). Version 4. Sinauer Associates, Sunderland, Massachusetts (2002).

[b37] Cunningham C. W. Can three incongruence tests predict when data should be combined? Mol. Biol. Evol. 14, 733–740 (1997). 9214746 10.1093/oxfordjournals.molbev.a025813

[b38] Xia X. DAMBE5: A comprehensive software package for data analysis in molecular biology and evolution. Mol. Biol. Evol. 30, 1720–1728 (2013). 23564938 10.1093/molbev/mst064 PMC3684854

[b39] Heled J. & Drummond A. J. Bayesian inference of species trees from multilocus data. Mol Biol Evol 27, 570–580(2010). 19906793 10.1093/molbev/msp274 PMC2822290

[b40] Akaike H. A new look at the statistical model identification. IEEE Trans. Automat. Control 19, 716–723 (1974).

[b41] Posada D. & Crandall K. A. Modeltest: testing the model of DNA substitution. Bioinformatics 14, 817–818 (2003). 9918953 10.1093/bioinformatics/14.9.817

[b42] Rambaut A., Suchard M. A., Xie D. & Drummond A. J. Tracer v.1.6. Available from http://beast.bio.ed.ac.uk/ tracer (Accessed: 27^th^ February 2016) (2014).

[b43] Rambaut A. FigTree 1.4.2. Tree Figure Drawing Tool (http://tree.bio.ed.ac.uk, Accessed: 27^th^ February 2016) (2014).

[b44] Magallón S., Gómez-Acevedo S., Sánchez-Reyes L. L. & Hernández-Hernández T. A metacalibrated time-tree documents the early rise of flowering plant phylogenetic diversity. New Phytol. 207, 437–453 (2015). 25615647 10.1111/nph.13264

[b45] Librado P. & Rozas J. DNASP v5: a software for comprehensive analysis of DNA polymorphism data. Bioinformatics 25, 1451–1452 (2009). 19346325 10.1093/bioinformatics/btp187

[b46] Yu Y., Harris A. J. & He X. J. RASP (reconstruct ancestral state in phylogenies): a tool for historical biogeography. Mol. Phylogen. Evol. 97, 46–69 (2015). 10.1016/j.ympev.2015.03.008 25819445

[b47] Bandelt H. J., Forster P. & Röhl A. Median-joining networks for inferring intraspecific phylogenies. Mol. Biol. Evol. 16, 37–48 (1999). 10331250 10.1093/oxfordjournals.molbev.a026036

[b48] Tajima F. Statistical method for testing the neutral mutation hypothesis by DNA polymorphism. Genetics 123, 585–595 (1989). 2513255 10.1093/genetics/123.3.585 PMC1203831

[b49] Fu Y. X. Statistical tests of neutrality of mutations against population growth, hitchhiking and background selection. Genetics 147, 915–925 (1997). 9335623 10.1093/genetics/147.2.915 PMC1208208

[b50] Excoffier L., Laval G. & Schneider S. ARLEQUIN, version 3.0: an integrated software package for population genetics data analysis. Evol.Bioinform. Online 1, 47–50 (2005). 19325852 PMC2658868

[b51] Harpending R. C. Signature of ancient population growth in a low-resolution mitochondrial DNA mismatch distribution. Human Biology, 66, 591–600 (1994). 8088750

[b52] Van Oosterhout C., Hutchinson W. F., Wills D. P. M. & Shipley P. Micro-Checker: software for identifying and correcting genotyping error in microsatellite data. Mol. Ecol. Notes 4, 535–538 (2004).

[b53] Goudet J. FSTAT: a program to estimate and test gene diversities and fixation indices (version 2.9.3). Available from http://www.unil.ch/izea/softwares/fstat.html (Accessed: 27^th^ February 2016) (2001).

[b54] Chapuis M. P. & Estoup A. Microsatellite null alleles and estimation of population differentiation. Mol. Biol.Evol. 24, 621–631 (2007). 17150975 10.1093/molbev/msl191

[b55] Rice W. R. Analyzing tables of statistical tests. Evolution 43, 223–225 (1989). 10.1111/j.1558-5646.1989.tb04220.x 28568501

[b56] Jombart T., Devillard S. & Balloux F. Discriminant analysis of principal components: a new method for the analysis of genetically structured populations. BMC Genet. 11, 94 (2010). 20950446 10.1186/1471-2156-11-94 PMC2973851

[b57] Wolfe K. H., Li W. H. & Sharp P. M. Rates of nucleotide substitution vary greatly among plant mitochondrial chloroplast, and nuclear DNAs. Proc. Natl. Acad. Sci. USA 84, 9054–9058 (1987). 3480529 10.1073/pnas.84.24.9054 PMC299690

[b58] Vigouroux Y. . Rate and pattern of mutation at microsatellite loci in maize. Mol. Biol. Evol. 19, 1251–1260 (2002). 12140237 10.1093/oxfordjournals.molbev.a004186

