# Supplementary material for: Colonization and diversification of the Euphorbia species (sect. Aphyllis subsect. Macaronesicae) on the Canary Islands
Source: Sci Rep. 2016 Sep 29;6:34454. doi: 10.1038/srep34454 (PMC5041082; doi:10.1038/srep34454)
Supplement: Supplementary Information [file srep34454-s1.pdf]

Scientific Reports

Supplementary Information

Article title

Colonization and diversification of the *Euphorbia* species (sect. *Aphyllis* subsect. *Macaronesicae*) on the Canary Islands

Authors

Ye Sun, Yanshu Li, Carlos Fabián Vargas-Mendoza, Faguo Wang, Fuwu Xing

Supplementary Figure S1. Bayesian phylogenetic tree of family Euphorbiaceae constructed based on DNA sequences of chloroplast tRNA-Leu, psbA-trnH, ndhF and nuclear ITS (see Table S1, Supporting Information for GenBank accessions). The posterior probability is shown above the branches. Blue bars represent confidence intervals of divergence time, which are shown to the right of internal nodes.

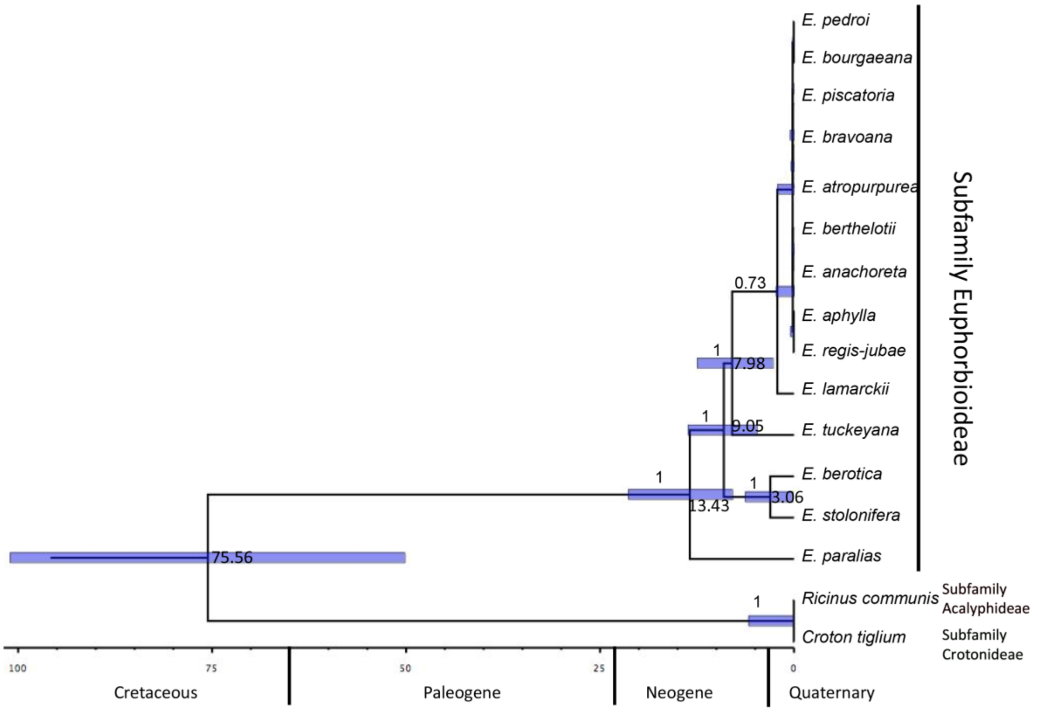

Supplementary Figure S2. Bayesian phylogenetic tree of sect. *Aphyllis* constructed based on DNA sequences of eight chloroplast regions and nuclear ITS (see Table S1, Supporting Information for GenBank accessions). The posterior probability is shown above the branches. Blue bars represent confidence intervals of divergence time, which are shown to the right of internal nodes.

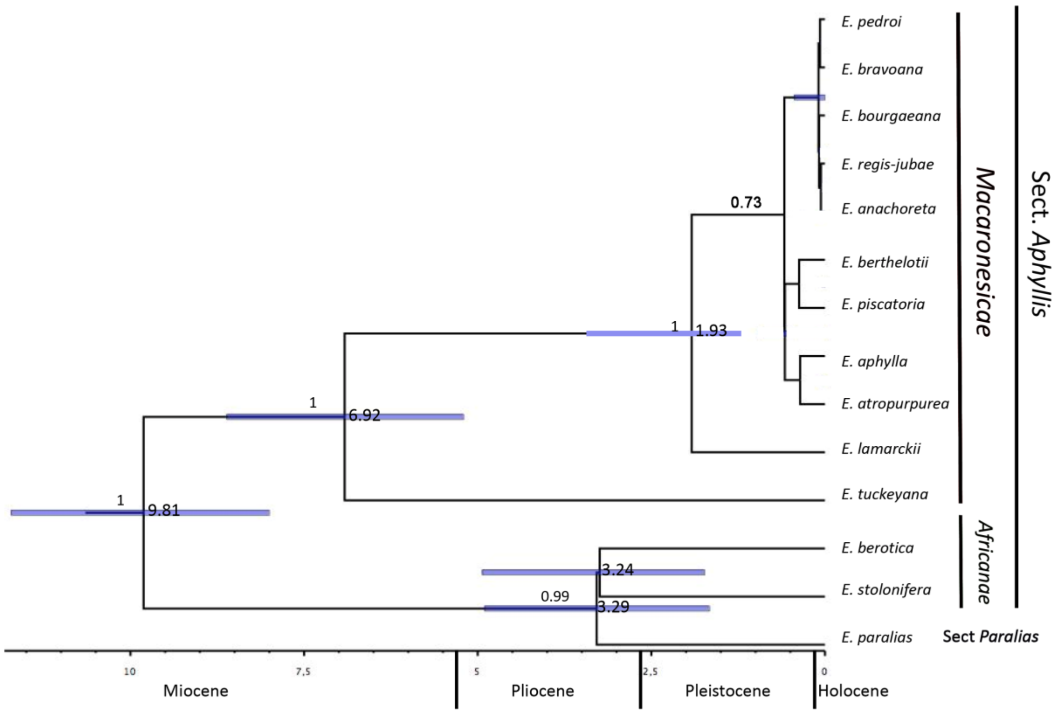

Supplementary Figure S3. Results of outlier test using ARLEQUIN software. There is no locus showed exceptionally high or low levels of population differentiation (F_{ST}), indicating no evidence showed that microsatellites are affected by selection.

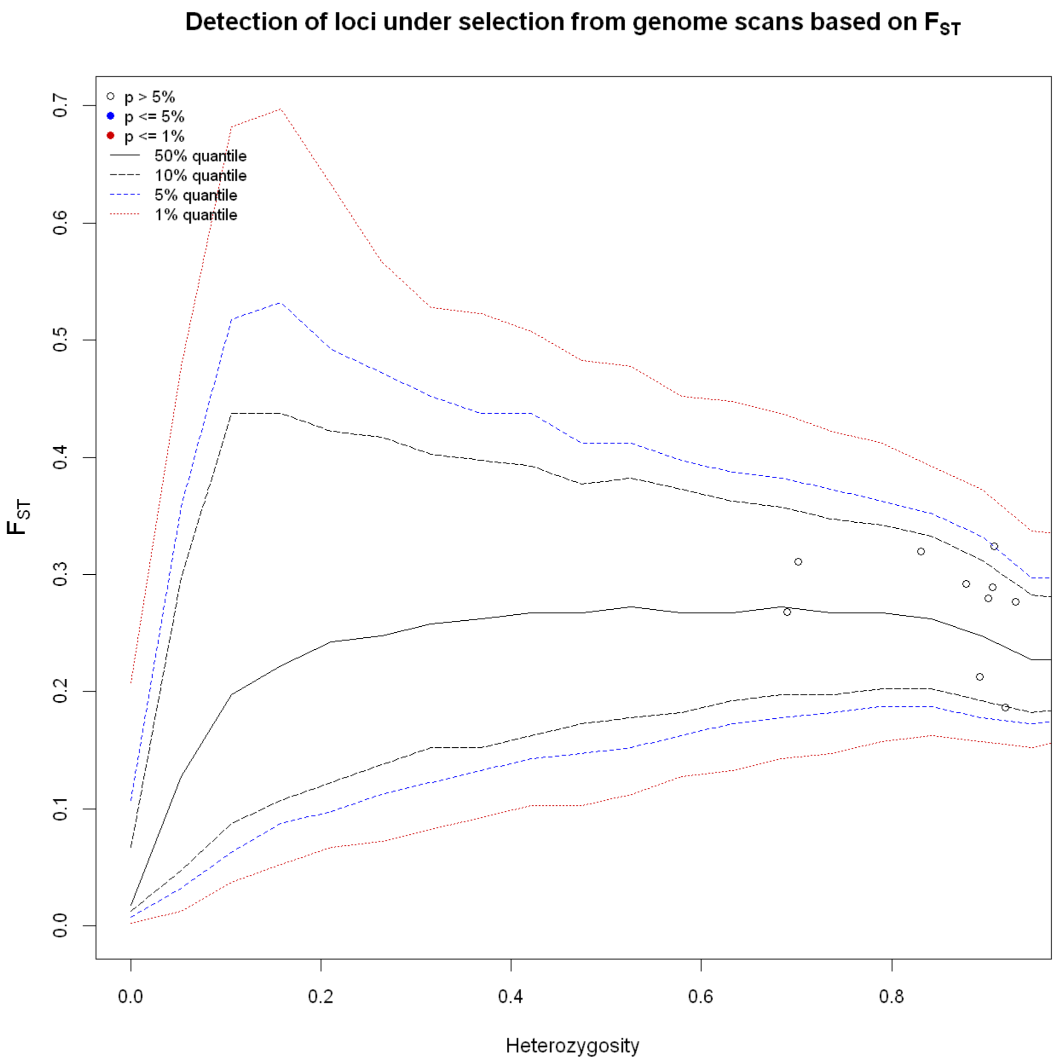

Supplementary Figure S4. The posterior probability distributions of migration rate estimated with IMa2. Curves are shown for the analysis between *E. lamarckii* and *E. atropurpurea* (a) and between *E. berthelotii* and *E. atropurpurea* (b).

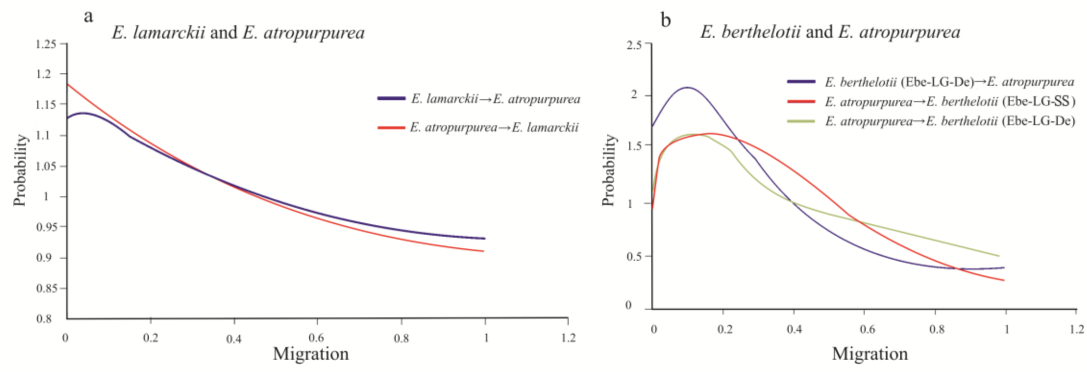

Table S1. GenBank Accession numbers of chloroplast and nuclear DNA sequences used for phylogenetic analysis of family Euphorbiaceae and sect. *Aphyllis*.

Species	cpDNA regions							
	tRNA-Ser	trnT-trnL	tRNA-Leu	psbA-trnH	tRNA-Lys (trnK)	tRNA-Gly	atpB-rbcL	ndhF
Subsect. *Macaronesicae*								
E. anachoreta	HQ900547.1	HQ900521.1	HQ900495.1	HQ900419.1	HQ900471.1	HQ900445.1	HQ900367.1	KC212440.1
E. aphylla	HQ900548.1	HQ900522.1	HQ900496.1	HQ900420.1	HQ900472.1	HQ900446.1	HQ900368.1	KC212445.1
E. atropurpurea	HQ900549.1	HQ900523.1	HQ900497.1	HQ900421.1	HQ900473.1	HQ900447.1	HQ900369.1	KC212445.1
E. berthelotii	HQ900551.1	HQ900525.1	HQ900499.1	HQ900423.1	HQ900475.1	HQ900449.1	HQ900371.1	EU022048.1
E. bourgaeana	HQ900553.1	HQ900527.1	HQ900501.1	HQ900425.1	HQ900477.1	HQ900451.1	HQ900373.1	KC212456.1
E. bravoana	HQ900554.1	HQ900528.1	HQ900502.1	HQ900426.1	HQ900478.1	HQ900452.1	HQ900374.1	EU022051.1
E. lamarckii	HQ900558.1	HQ900532.1	HQ900506.1	HQ900430.1	HQ900481.1	HQ900456.1	HQ900378.1	KC212553.1
E. pedroi	HQ900565.1	HQ900539.1	HQ900513.1	HQ900437.1	HQ900487.1	HQ900463.1	HQ900385.1	KC212596.1
E. piscatoria	HQ900566.1	HQ900540.1	HQ900514.1	HQ900438.1	HQ900488.1	HQ900464.1	HQ900386.1	
E. regis-jubae	HQ900567.1	HQ900541.1	HQ900515.1	HQ900439.1	HQ900489.1	HQ900465.1	HQ900387.1	KC212617.1
E. tuckeyana	HQ900571.1	HQ900545.1	HQ900519.1	HQ900443.1	HQ900493.1	HQ900469.1	HQ900391.1	KC212667.1
Subsect. *Africanae*								
E. berotica	HQ900550.1	HQ900524.1	HQ900498.1	HQ900422.1	HQ900474.1	HQ900448.1	HQ900370.1	KC212451.1
E. stolonifera	HQ900569.1	HQ900543.1	HQ900517.1	HQ900441.1	HQ900491.1	HQ900467.1	HQ900389.1	KC212644.1
Sect. Paralias								
E. paralias		JN009976.1	JN249696.1				JN891117.1	KC212595.1
Subfamily Crotonoideae and Acalyphoideae								

Croton tiglium			KP878454.1	KR534132.1				
Ricinus communis			AY794734.1	JQ279716.1				FJ670089.1

Species	Nuclear
	ITS1 and ITS2
Subsect. *Macaronesicae*	
E. anachoreta	KC212173.1
E. aphylla	AF537540.1
E. atropurpurea	AF537542.1
E. berthelotii	HQ900585.1
E. bourgaeana	JN250121.1
E. bravoana	HQ900588.1
E. lamarckii	HQ900619.1
E. pedroi	KC212332.1
E. piscatoria	HQ900644.1
E. regis-jubae	AF537541.1
E. *tuckeyana*	HQ900663.1
Subsect. *Africanae*	
E. berotica	HQ900584.1
E. stolonifera	HQ900658.1
Sect. Paralias	
E. paralias	JN250207.1

Subfamily Crotonoideae and Acalyphoideae	
Croton tiglium	KP878399.1
Ricinum communis	KJ000402.1

Supplementary Table S2. The maximum-likelihood estimates (MLE) and 95% highest probability density (HPD) of gene flow rate. The populations correspond to the names in Table 1.

Species and populations	Gene flow from Population i to Population j	MLE	Lower bound of 95% HPD	Higher bound of 95% HPD
E. lamarckii				
Ela-T-Me	Ela-T-Me ->Ela-T-An	0.9895	0.0015	0.9895
	Ela-T-Me ->Ela-LG-Ag	0.0005	0.0000	0.9360
	Ela-T-Me ->Ebe-LG-SS	0.0005	0.0000	0.9331
	Ela-T-Me ->Ebe-LG-De	0.0005	0.0000	0.9331
	Ela-T-Me -> Eat-T-Ma	0.0312	0.0000	0.9351
Ela-T-An	Ela-T-An ->Ela-T-Me	0.9895	0.0000	0.9895
	Ela-T-An ->Ela-LG-Ag	0.0005	0.0000	0.9390
	Ela-T-An ->Ebe-LG-SS	0.0005	0.0000	0.9331
	Ela-T-An ->Ebe-LG-De	0.0005	0.0000	0.9331
	Ela-T-An -> Eat-T-Ma	0.0005	0.0000	0.9360
Ela-LG-Ag	Ela-LG-Ag ->Ela-T-Me	0.0005	0.0000	0.9360
	Ela-LG-Ag ->Ela-T-An	0.0005	0.0000	0.9351
	Ela-LG-Ag ->Ebe-LG-SS	0.0005	0.0000	0.5737
	Ela-LG-Ag ->Ebe-LG-De	0.0005	0.0000	0.5678
	Ela-LG-Ag -> Eat-T-Ma	0.0005	0.0000	0.6470
E. berthelotii				
Ebe-LG-SS	Ebe-LG-SS ->Ela-T-Me	0.0005	0.0000	0.9311
	Ebe-LG-SS ->Ela-T-An	0.0005	0.0000	0.9331
	Ebe-LG-SS ->Ela-LG-Ag	0.0005	0.0000	0.4480
	Ebe-LG-SS ->Ebe-LG-De	0.0005	0.0000	0.9182
	Ebe-LG-SS -> Eat-T-Ma	0.0045	0.0000	0.6707
Ebe-LG-De	Ebe-LG-De ->Ela-T-Me	0.0005	0.0000	0.9341
	Ebe-LG-De ->Ela-T-An	0.0005	0.0000	0.9331
	Ebe-LG-De ->Ela-LG-Ag	0.0005	0.0000	0.5242
	Ebe-LG-De ->Ebe-LG-SS	0.9895	0.0015	0.9895
	Ebe-LG-De -> Eat-T-Ma	0.1025	0.0000	0.8677
E.atropurpurea				
Eat-T-Ma	Eat-T-Ma ->Ela-T-Me	0.0005	0.0000	0.9341
	Eat-T-Ma ->Ela-T-An	0.0005	0.0000	0.9321
	Eat-T-Ma ->Ela-LG-Ag	0.0005	0.0000	0.6163
	Eat-T-Ma ->Ebe-LG-SS	0.1836	0.0000	0.8390
	Eat-T-Ma ->Ebe-LG-De	0.1262	0.0000	0.8955
